# Dimensionality reduction techniques in pupillometry research: A primer for behavioral scientists

**DOI:** 10.3758/s13428-025-02786-0

**Published:** 2025-11-10

**Authors:** Serena Castellotti, Irene Petrizzo, Roberto Arrighi, Elvio Blini

**Affiliations:** 1 Department of Translational Research on New Technologies in Medicine and Surgery, University of Pisa, Pisa, Italy; 2 Department of Neuroscience, Psychology, Pharmacology and Child Health, University of Florence, Florence, Italy; 3 Centre for Mind/Brain Sciences, University of Trento, Rovereto, Italy; 4 Via di San Salvi 12, Building 26, 50135 Firenze, Italy

**Keywords:** Pupillometry, Dimensionality reduction, Principal components analysis, Pupillary manifold, Autonomic nervous system, Cognitive load, Pupillary light reflex

## Abstract

The measurement of pupil size is a classic tool in psychophysiology, but its popularity has recently surged due to the rapid developments of the eye-tracking industry. Concurrently, several authors have outlined a wealth of strategies for tackling pupillary recordings analytically. The consensus is that the “temporal” aspect of changes in pupil size is key, and that the analytical approach should be mindful of the temporal factor. Here we take a more radical stance on the matter by suggesting that, by the time significant changes in pupil size are detected, it is already *too late*. We suggest that these changes are indeed the result of distinct, core physiological processes that originate several hundreds of milliseconds *before* that moment and altogether shape the observed signal. These processes can be recovered indirectly by leveraging dimensionality reduction techniques. Here we therefore outline key concepts of temporal principal components analysis and related rotations to show that they reveal a latent, low-dimensional space that represents these processes very efficiently: a pupillary manifold. We elaborate on why assessing the pupillary manifold provides an alternative, appealing analytical solution for data analysis. In particular, dimensionality reduction returns scores that are (1) mindful of the relevant physiology underlying the observed changes in pupil size, (2) extremely handy and manageable for statistical modelling, and (3) devoid of several arbitrary choices. We elaborate on these points in the form of a tutorial paper for the functions provided in the accompanying R library “Pupilla.”

## Introduction

Physiological measures have been crucial in the development of cognitive and behavioral sciences. They allow researchers to track cognitive processes unfolding over time, revealing, e.g., whether these processes are roughly homogeneous or composed of distinct components. As such, they offer invaluable mechanistic insights beyond traditional behavioral indices such as accuracy and response times. In many instances, however, assessing physiological reactions represents more than yet another methodological tool: it is a way to skim behavioral variability, e.g., motor or perceptual noise, to better isolate and quantify a theoretical construct of interest. For example, physiological (body) reactions are very well suited to reflect affect because they are at the core of its definition (Damasio, 1996). Fear and anxiety, for instance, are explicitly defined in terms of the associated autonomic reactions (De Zorzi et al., 2021; Grupe & Nitschke, 2013). This, however, also applies to more abstract concepts. Cognitive load, for example, can be defined in terms of the (absolute) amount of information to be processed at once, but the very same “amount” will inevitably impact individuals differently. One way to resolve this issue is to design adaptive or psychometric procedures (Blini et al., 2025; Bonato et al., 2010, 2019). Alternatively, one can resolve to more physiologically oriented definitions of cognitive load, such as the amount of (autonomic) activation that reflects the mental effort required to accomplish a task (e.g., pupil dilation; Blini et al., 2024a, b; Hess & Polt, 1964). The point of these examples is that, in most cases, the interest of using physiological techniques lies precisely in the possibility to isolate specific physiological processes.

Unfortunately, many common techniques may not provide sufficient depth or channels to easily discriminate between different generative processes. It is the case for classic psychophysiological measures, including electrodermal activity, heart rate, and pupillometry, which are based on recordings from a single, continuous channel. This feature limits the precision by which distinct components can be recovered, because multiple processes as well as multiple sources of noise are mixed and become hardly distinguishable. The struggle consists in recovering the latent (unobserved) physiological processes indirectly, based on their joint impact on the signal itself. In the case of heart rate, for example, several computational approaches have been developed as a consequence, capable of reflecting different aspects of autonomic processing and of returning less simplistic and clinically more useful indications (Armstrong et al., 2022). In this article, we focus, however, on pupillometry, which is perhaps less common than other psychophysical, peripheral activation techniques, but is nonetheless one of the elective tools of behavioral neuroscience. Pupillometry has been used to investigate a wide range of mental processes as detailed in several recently published reviews (Banks et al., 2015; Binda & Murray, 2015a; Castellotti et al., 2025; Einhäuser, 2017; Koevoet et al., 2024; Laeng et al., 2012; Mathôt, 2018; Mathôt & Van der Stigchel, 2015; Sirois & Brisson, 2014; Strauch et al., 2022; Vilotijević & Mathôt, 2023). Alongside the renewed interest in this technique, we have also witnessed a surge in methodological papers aimed at suggesting how to tackle pupillary recordings analytically (Attard-Johnson et al., 2019; Cai et al., 2024; Calignano et al., 2024; Fink et al., 2024; Hershman et al., 2019, 2023; Mathôt & Vilotijević, 2023; van Rij et al., 2019). Each proposed method has advantages, pitfalls, and a specific range of application. For example, assessing pupillary recordings in free-viewing, naturalistic situations requires much different cautions than assessing pupil size in a strictly controlled experimental setting. Here we focus on the latter scenario, in which it is arguably easier to extract physiologically meaningful parameters: indeed, laboratory settings are generally carefully controlled for potential confounds, and experimental conditions typically vary along one dimension.

We suggest that any analytical approach, prior to statistical considerations, should first and foremost be mindful of the physiological processes that are probed by the technique at hand. Only by clearly identifying the relevant constraints imposed by the underlying biology can one, with the aid of appropriate tools, safely dissect the signal into multiple sources and processes. More generally, it is unfortunate to assess physiological data, as *any other data*, that are detached from their physiological meaning. Here we elaborate on these points in the form of a tutorial paper for the functions provided in the accompanying R library “Pupilla” (https://eblini.github.io/Pupilla/). Before diving into methodological details, however, we will review some key physiological notions that underlie changes in pupil size, because these are precisely the relevant constructs that we should strive to recover analytically.

## Core physiological notions

The core task of human pupils is to manage the amount of light reaching the retina (Campbell & Gregory, 1960; Loewenfeld, 1999; Mathôt, 2018, 2020; Vilotijević & Mathôt, 2023). Dilated pupils enhance visual sensitivity for dim and/or peripheral stimuli as they allow more light to reach the retina; small pupils protect from excessive light and favor central acuity (Mathôt, 2020; Mathôt & Van der Stigchel, 2015). Pupil size is, however, also a privileged window into core autonomic processes.

The autonomic nervous system (ANS) regulates the body functions (mostly) outside of voluntary control, and it is divided into two components. The parasympathetic branch is involved in homeostatic processes that promote relaxation, e.g., a slower heart rate, and is associated with small pupils. The sympathetic branch promotes behavioral activation and is instead associated with increased heart rate and large pupils. Under controlled light levels, therefore, the diameter of the pupils reflects the relative autonomic balance—i.e., the individual’s position along the relaxation–activation continuum. This measure reflects a “tonic” functioning mode of the pupil, in the sense that it is the result of relatively stable and long-lasting physiological states (Pelagatti et al., 2025). However, in experimental settings, we typically focus on dilation/constriction events that occur at a much quicker pace (the “phasic” mode). These event-related changes can be systematically observed once the baseline pupil fluctuations are properly controlled for (i.e., via baseline subtraction). Phasic responses can also be grouped into constriction events (primarily responses to light) and dilation events (including a number of psychosensory/cognitive responses) (Mathôt, 2018), each traditionally associated with the two distinct branches of the ANS, and each providing valuable insights about cognitive processes.

Indeed, even responses to light cannot be regarded as purely reflexive, since an increasing bulk of literature has highlighted how they can be modulated by, e.g., attentional processes or brightness illusions (Binda et al., 2013; Binda & Murray, 2015b; Blini & Zorzi, 2023; Castaldi et al., 2021; Castellotti et al., 2020; Mathôt et al., 2013). Pupil dilation can occur for all sorts of arousing, demanding, or affectively connoted tasks and stimuli (Bradley et al., 2008; Castellotti, Francisci, et al., 2021a, b; Castellotti, Scipioni, et al., 2021a, b; de Winter et al., 2021; Dureux et al., 2021; Finke et al., 2021; Hess & Polt, 1960). Altogether, these changes reflect dynamic shifts between attentional states as instantiated by the locus-coeruleus noradrenaline system, and are crucial for adaptive behaviors (Aston-Jones & Cohen, 2005). Unfortunately, these responses are not mutually exclusive, and they almost invariably happen simultaneously, at the onset of any salient stimulus change, though with slightly different latencies. As a result, even if one process may be particularly prominent in a given task, the pupil traces inevitably return a combination of numerous, sometimes opposed sources. Since pupil size is only represented by a single channel, it is hard to determine whether increased pupil dilation is the result of increased sympathetic activity or rather parasympathetic inhibition. It has been suggested that both these physiological processes are well represented in pupil dilation to mental effort: dilation would occur at first due to cortical inhibition of the parasympathetic efferent pathway, particularly so in bright environments; then, later in time and more vigorously, pupils would dilate due to a primarily sympathetic activation. These observations were drawn from both carefully designed pharmacological interventions and the manipulation of environmental light levels (Steinhauer et al., 2004; Steinhauer & Hakerem, 1992). Most key notions were however already well known by the late nineteenth century (as reviewed by Loewenfeld & Lowenstein, 1993; Strauch, 2024).

The neuronal correlates of pupil dynamics reveal a very heterogeneous picture, with modulations of large-scale resting-state networks and local population activity within more specialized ones (Joshi et al., 2016; Pfeffer et al., 2022; Podvalny et al., 2021; Radetz & Siegel, 2022; Viglione et al., 2023; Wang & Munoz, 2015). Interestingly, Radetz and Siegel (2022) recently identified, using magnetoencephalography (MEG), three networks which either caused or followed changes in pupil size: a widespread prefrontal network with activity predominantly in the theta band, a precentral network predominantly expressed in the beta band, and an occipitoparietal network at frequencies of around 16 Hz. This multifaceted picture was not apparent when using classic correlational analysis (i.e., correlating network activity with pupil size or its derivative for each time point). Rather, most studies of this sort employed a cross-correlation approach, that is, they assessed how correlations between neuronal activity and pupil size changed at varying lags. The networks mentioned above presented peak lags between 0.2 and 1.2 s, suggesting that these processes may start several hundreds of milliseconds *before* a given pupil diameter is observed. We maintain that reconstructing these source signals bears much greater interest than assessing their indirect results and/or the resulting trajectory of changes.

## Analytical approaches for pupillometry research: Past and present

One classic strategy to tackle the analysis of pupil data is to define a window of interest from which to extract summary statistics. For example, the mean, median, or maximum dilation values can be extracted from a given time window, and then submitted to an inferential technique of choice (e.g., analysis of variance [ANOVA] or *t*-tests). There are several known problems with this approach. The most compelling one is probably that how a time window is chosen can be arbitrary, inconsistent within and between research groups. To some extent, this limit can be overcome by leveraging several data-driven approaches. Computing the first derivative—which represents the velocity of pupil size changes over time—can for example precisely identify constriction/dilation events and peaks, and thus inform the choice of time window (Joshi et al., 2016; Pelagatti et al., 2025; Ten Brink et al., 2024). Overall, however, the problems with resolving to single values can be summarized by the following critique: the temporal aspects of changes in pupil size, i.e., their dynamics, are insufficiently considered. Thus, more recent developments in analytical approaches are mindful of the factor “time” in the analysis of pupil data (Sirois and Brisson, 2014). The advantages of this approach are well illustrated by Hershman et al. (2023). Their main argument is that the pupils do not suddenly change their size from one time point to another. Rather, pupil size and differences between conditions build up gradually and slowly, and assessing their time course as well as their *persistence* in a temporal perspective is likely to provide a more precise picture of pupillary dynamics.

To account for the “time” factor, several approaches have been put forward. The simplest one is probably data binning: here, pupil traces are divided into several time windows, covering the entire time course of the trial. This approach still involves obtaining summary statistics from these bins, but provides at the very least much more granularity and sensitivity to temporal dynamics. More advanced, contemporary methods (Sirois and Brisson, 2014) include, e.g., linear mixed-effects models (Baayen et al., 2008; Bates et al., 2015; Mathôt & Vilotijević, 2023). This approach is particularly powerful: the factor “time” can be accounted for without excessive downsampling of the data, and with enhanced flexibility to specify an optimal matrix of random effects (but see Barr et al., 2013; Blini et al., 2018, 2020; Matuschek et al., 2017; Scandola & Tidoni, 2024). Known limitations of these approaches are essentially the intensive usage of computational power and, more compelling, the potentially very high number of statistical comparisons which, if not controlled for, may inflate the rate of false positives. Several strategies can obviate this issue, such as cluster-based permutation tests (Blini & Zorzi, 2023), cross-validation (Mathôt & Vilotijević, 2023), corrections for the false discovery rate (FDR, Benjamini & Hochberg, 1995), or Bayesian approaches (Hershman et al., 2019, 2023). More advanced methods are also available, for example, exploiting general additive mixed models (van Rij et al., 2019). These models elegantly account for potentially nonlinear effects by introducing smoothing functions that can better capture the *trajectory* of changes in pupil size. This increased power however comes at the cost of adding several layers of complexity, arbitrary choices, and ultimately a more difficult interpretation of the results (but see van Rij et al., 2019).

Here we fully endorse the argument by Hershman et al. (2023) that the temporal dynamics of the pupils are key. Actually, we take a more radical stance on this argument by suggesting that, once differences between conditions emerge, *it is already too late*. As outlined in the introduction, at this point, the latent processes that have generated them in the first place are already full-blown. Ironically, however, defending this position leads us to break a spear for a single (or few) value(s) approach, one that happens to be more mindful of the underlying physiology.

## Towards more physiologically meaningful features with dimensionality reduction

This tutorial aims to illustrate a few essential concepts of dimensionality reduction applied to pupillometry research. We will do so by leveraging R functions (R Core Team, 2023) that are made freely available from GitHub (https://github.com/EBlini/Pupilla). The installation requires the R package remotes. The following step must be taken only the first time that the package is installed; however, given that the package is under continuous development, we suggest repeating this step regularly.
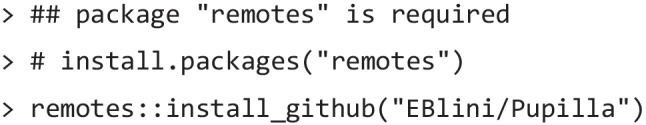


This passage will also install all the dependencies needed for this tutorial. After installation, the package will be made available for use, similarly to other R packages.
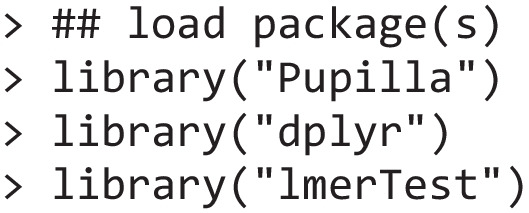


**Fig. 1:**
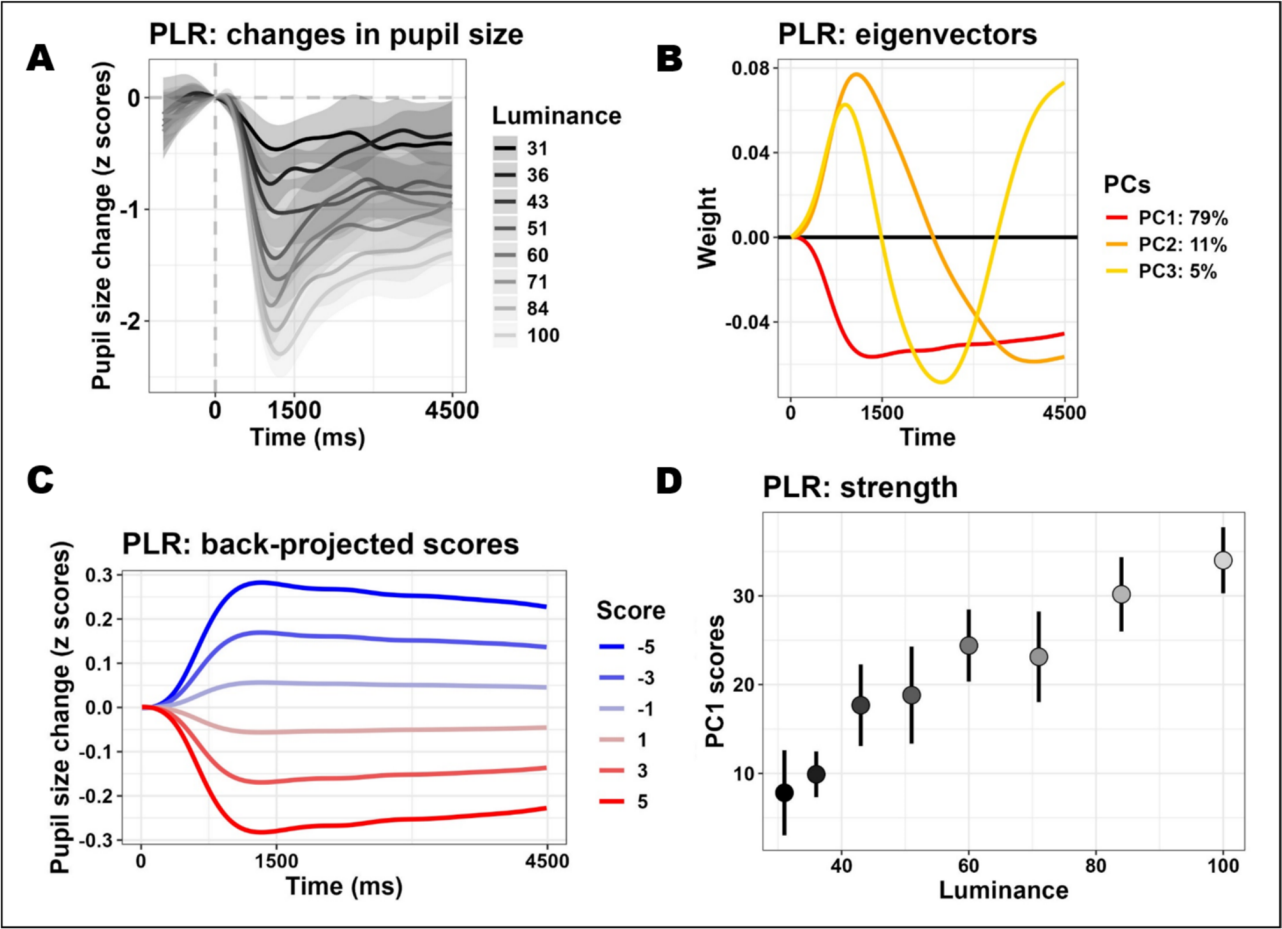
Panel **A** depicts the mean (95% confidence interval) changes in pupil size when participants were passively looking at numbers changing luminance levels. The panel therefore depicts the classic pupillary response to light (PLR). When temporal principal components analysis (PCA) is performed on these traces, three components can describe about 95% of the overall variability. The eigenvectors in panel **B** illustrate the relationship between the original variables (the time points) and the three components. The eigenvector of the first component explains most of the original variability and, more importantly, presents a shape that effectively captures our principled notion of how the PLR is supposed to unfold in time: a rather quick, robust constriction of the pupils followed by pupillary escape. Thanks to the three components, the data in panel **A** can be faithfully described by three values (per trace), the scores, with only a minimal loss of accuracy. The temporal structure of the components (panel **B**) gives meaning to what the scores represent: the overall alignment of the trial with this shape, the “strength” of the component. For example, in panel **C**, we choose a few PC1 scores and depict the implied temporal structure, to illustrate how scores are embedded with temporal information—this is, however, only given to illustrate the concept, since in theory the same score can actually be obtained from multiple combinations of the original data. Panel **D** is an example of how analyses can be greatly simplified by assessing PC scores. The scores of PC1, for example, can be taken to reflect the strength of PC1 (the PLR) in a different coordinate system composed of fewer dimensions: Panel **D** shows that different luminance conditions are indeed very well separable in this latent space (error bars represent 95% confidence intervals)

To illustrate how dimensionality reduction can be applied to pupillometry, we will use data from our lab as an example (Blini et al., 2024b). Here, healthy young participants were simply exposed to different light levels. They were viewing a matrix of numbers superimposed on a black background: nine “zeros” were arranged in three rows and their color intermittently changed (shade of gray), so that we could map phasic responses to eight different luminance levels (Fig. 1A). Thus, the data highlight the well-known pupillary light reflex (PLR).

If Pupilla is installed with the settings above, these data will be included as well. It is very straightforward to load the data in the current environment.
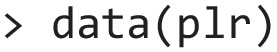


The command above imports a dataframe named plr in the current environment. The dataframe is in R’s long format (i.e., one row per sample/time point) and includes the following variables:

- Subject simply marks different participants in the experiment (*N* = 20).
- Trial denotes different trial repetitions within each participant.
- Time denotes the different timestamps within each trial. Time, in this case, is negative during the presentation of a baseline condition; the value “0” corresponds to the presentation of the target, which lasts on screen 4,500 ms.
- Luminance denotes different levels of our experimental manipulation. Thus, this is the independent variable in the example.
- Pupil denotes pupil size at the specific time point. Thus, this is the dependent variable in the example.

The data at this point have already been preprocessed (Blini et al., 2024a, b). In particular, blinks and artifacts were removed, pupil size was transformed into *z*-scores, and for each entry we subtracted a baseline value obtained from a 500-ms window centered on the presentation of the targets. These steps are all very common, if not mandatory, when the goal is to analyze phasic changes in pupil size; we refer to other excellent tutorials for a more detailed overview of all important steps (Mathôt & Vilotijević, 2023). Pupilla does contain utility functions for these steps, which are not the focus of this tutorial, and we refer the reader to the manual and vignettes for their use: https://eblini.github.io/Pupilla/index.html.

### Temporal principal components analysis (PCA)

A complete, mathematical overview of PCA is beyond the scope of this article. At its core, however, PCA works by creating n uncorrelated (orthogonal) variables that progressively account for a decreasing amount of the variance in the original dataset (Jolliffe & Cadima, 2016). In other words, these variables (the principal components) are linear combinations of the original ones that are capable of preserving as much of the original variability as possible. One should ideally be able to recreate the original dataset, or a reasonably good approximation thereof, with very few principal components. The more the original variables are correlated, the easier this task is, as their covariance is ultimately what lies at the core of PCA. Hence, the relevance of this technique for pupillometry: the number one predictor of pupil size at a given moment is pupil size in a previous moment, i.e., the signal is strongly autocorrelated across time points.

Temporal PCA in Pupilla is implemented as reduce_PCA(), a wrapper around stats::prcomp() (R Core Team, 2023), and is very straightforward (once the data have been preprocessed).
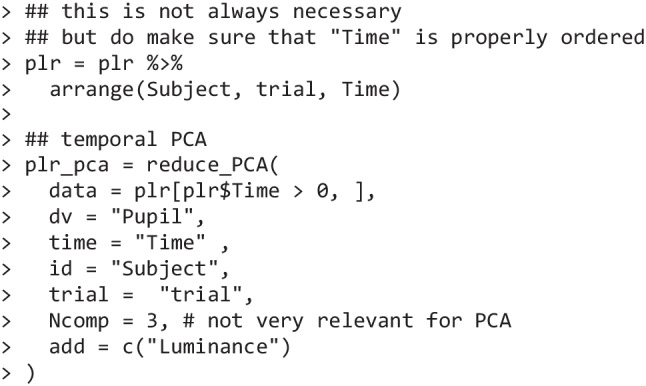


Note that only time points after the target presentation have been included in the data parameter. This is to avoid the inclusion of time points with zero variance, which would make further analyses impossible. Aside from this, analyses typically cover the entire duration of the trial, which is established according to one’s experimental questions and needs (in our case, 4.5 s). Also note that there are no missing values in the data. Trials with missing samples must be interpolated or discarded beforehand; the presence of missing values will cause the omission of the entire trial from the analyses. In the remaining parameters, the user must only specify the following:

- dv,time,id,trial: the labels used to indicate the dependent and grouping variables.
- add: a character vector indicating the name of the independent variables. These variables will be conveniently appended to the final (reduced) dataframe.
- Ncomp: the number of components to retain. Because temporal PCA is deterministic, this is only useful to have clearer results. Another option is to return all the components that are necessary to explain at least some percentage of the total variance (when Ncomp < 1, e.g., Ncomp = 0.95 will select the number of components needed to explain at least 95% of the overall variability).

It is useful to note that a complete description of the parameters and results of this call can be accessed offline with the usual notation ?reduce_PCA.

In this example, each time point was used as a standalone variable; each trial from each individual (though averages would have been appropriate as well) was instead used as an observation. In light of the strong autocorrelation of the data, as expected, we found that three components were sufficient to reconstruct 94.4% of the initial data. The amount of variability along a given direction (and thus the order of the components) is technically indexed by and proportional to the corresponding eigenvalues. Eigenvalues are reported (square-rooted) in the first line of the summary presented below, and are accompanied by the share of variance in the data that they can account for.
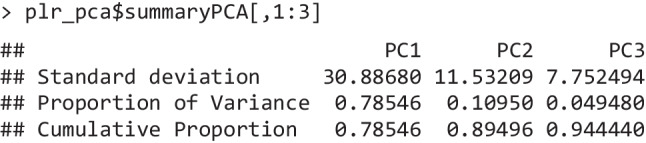


The three principal components are derived from the eigenvectors (Fig. 1B), stored in the entry Loadings of the returned object (plr_pca$Loadings). The eigenvectors identify the directions (with respect to the original variables) along which the original data vary the most. In our case, they illustrate the relationship between the time points and the components. Note that the overall sign of the eigenvector is arbitrary: multiplying one eigenvector by −1 would provide a mathematically equivalent solution. There are several helper functions to depict the eigenvectors. In the specific case of models with three components, one can use plot_fingerprints() (Fig. 1B).
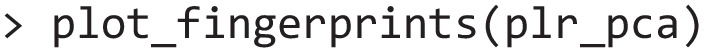


In the context of these results, the conclusion is that we can distill each trial from 450 values (one per time point) to only three values, with only a minimal loss of accuracy (≈ 5% of the overall variability): these values are the *scores*. Indeed, the original data (or new data with compatible dimensions) can be projected onto the new, low-dimensional coordinate system defined by the eigenvectors. One can think of scores as the weighted average of pupil size at all time points, whereby the weighting factor is given by the eigenvectors. For example, in Fig. 1C, we back-project possible score values for the first component (and the first component alone) in the original space. The scores indeed summarize the temporal information conveyed by the eigenvectors: their temporal structure gives meaning to what the scores represent, that is, the overall alignment of the trial with the component’s shape (its “strength”).

The most common scenario after PCA is therefore to assess the scores (plr_pca$Scores). For example, one can start by appreciating the fact that the first component explains a very large share of the variability in the data (79%) and, most importantly, its eigenvector reflects our informed notion of what the pupillary response to light should be: a rather quick constriction of the pupils which peaks within 1–1.5 s and then gradually recoups (or “escapes,” Mathôt, 2018). The scores for this component may therefore be taken to represent, as a consequence, the “strength” of the PLR on a latent, low-dimensional space. In order to test the independent variables, in our case luminance level, one can use the statistical model of choice, e.g., linear mixed effects models (Bates et al., 2015; Kuznetsova et al., 2017), directly on the scores.
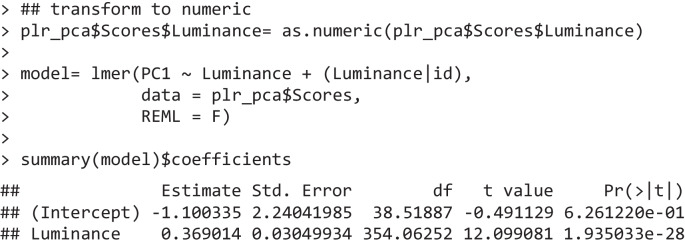


The scores (and eigenvectors) are indeed returned as a dataframe amenable to all sorts of further processing via R’s built-in functions. The scores dataframe now has one row per trial, and as many columns as there are components (in addition to the grouping variables). In Fig. 1D, the mean scores are depicted as a function of the luminance that generated them; one can indeed appreciate that the scores map very well onto different luminance levels, as suggested by the inferential test above.

There are advantages and pitfalls in using this approach. The first advantage is that it returns single (or few) values. Single values are much handier to analyze with the statistical model of one’s choosing (one example is given in Fig. 1D). Unlike other summary functions, the scores obtained in this way are to some extent mindful of the temporal aspect. The temporal dynamics are illustrated by eigenvectors. As mentioned, these variables show the relative importance and contribution of each time point to a component. This contribution is never dichotomic (i.e., is either significant or not); rather, it is graded, with time points that yield a negligible contribution and time points that are highly influential, including one peak timepoint. Hence, to reiterate, one can think of scores as the weighted average of pupil size at all these time points. The peak time point will contribute the most, but will not be the only determinant of the scores. In short, scores are embedded with the temporal information conveyed by the eigenvectors, and thus index the relative strength of one component in the data. Finally, the approach dispenses with choosing a more or less arbitrary time window, being completely data-driven. Thus, one major cause of inconsistencies between studies can be solved elegantly. This is not only a practical feature related to simplification. One may argue that components beyond the first few (e.g., explaining a very residual part of the initial variability) may gradually become less interesting, capturing not the most relevant, quintessential pupil signal but rather some source of measurement noise or artifact. On the contrary, resolving to the first principal components can be considered as a way to skim the overall variability of the signal to reach more meaningful proxy values of an underlying process. Hershman et al. (2023) also stressed the importance of the persistence of an effect. We maintain that assessing the strength of a component through PCA scores is a sound way to assess persistence precisely because any effect is expected to have graded contributions to pupil size at different time points; judging persistence by, e.g., whether an effect lasts longer (results in more significant time points) instead loses this nuance, even though it may reach similar conclusions.

For example, we can compare the results of this analysis with more established temporal methods, both available in Pupilla as lmem_test() and bayesian_test().

The first implements, for each time point, a linear mixed-effects model (LMEM) as powered by lme4 (Bates et al., 2015; Kuznetsova et al., 2017). LMEMs are both powerful—leveraging all observations rather than just averages—and flexible, as they allow for the modeling of complex experimental designs, including random slopes and hierarchical structures (Baayen et al., 2008). This function is seemingly more precise in locating a cluster of significant effects of luminance on pupil size between 440 and 4,500 ms.
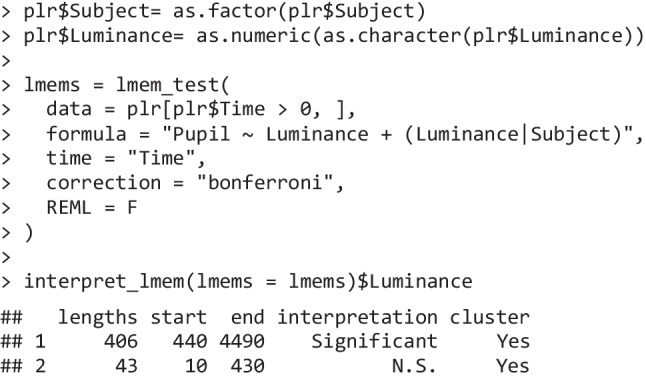


The Bayesian counterpart reaches similar conclusions. For example, bayesian_test is a wrapper around BayesFactor::generalTestBF() (Morey & Rouder, 2024) and similarly performs a linear model for each time point, returning a Bayes factor as an inferential criterion. The priors used are standardized, Cauchy-distributed priors (Morey & Rouder, 2011) which have been widely adopted, making this approach roughly in line with Hershman et al. (2019). Even in this case, temporal analysis reveals a cluster of time points that likely see an impact of luminance manipulations.
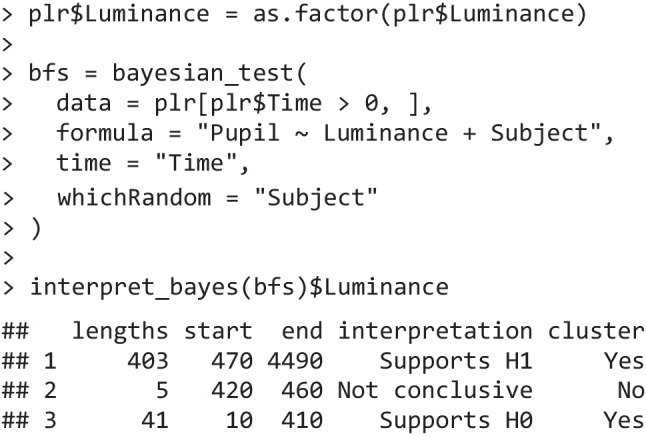


The precision in the estimation of the time window strictly depends on how conservative our threshold for statistical significance is. Generally speaking, we understand how having a precise number for the time in which an effect reaches significance may be comforting for many colleagues. However, we also believe that this reasoning may implicitly perpetuate an idea of on/off effects on pupil size, whereas the underlying processes are graded in nature. Furthermore, performing these tests in a batch significantly limits our ability to assess model performance and convergence. Convergence has been discussed at length as a key issue in sophisticated statistical models, with many authors suggesting adapting our models’ specifications to the data, while others suggest keeping them maximal (Barr et al., 2013; Blini et al., 2018, 2020; Matuschek et al., 2017; Scandola & Tidoni, 2024). The preliminary extraction of one relevant score, instead, allows one to fine-tune the approach to statistical modelling, for example by specifying the matrix of random effects that is most appropriate for the data. In several use cases, from these significant intervals, one summary statistic must be extracted for other purposes anyway. For example, imagine that the ultimate goal is to obtain normative data in a sample of healthy participants to provide a benchmark for a clinical population. One possibility is to use the average signal within the time window. Alternatively, one can resolve directly to the scores computed above, because they already provide an accurate summary of the signal’s overall shape.

In summary, both these approaches—i.e., temporal analysis with established methods or via preliminary data reduction—mostly converge in returning interpretable and useful indications. Methods based on PCA, however, have been used before to describe the major component(s) of pupil traces (Young & Kennish, 1993), including that of the PLR (Binda & Murray, 2015b). Previous studies have indeed attempted to link different components to specific functions, for example a sustained response (the PLR) versus more residual responses (e.g., transient responses, cognitive variables, accommodation-related phenomena) (Binda & Murray, 2015b; Young & Kennish, 1993). In other words, the intuition was that different components may have a specific functional interpretation. However, in the case of tasks that elicit multiple, heterogeneous processes (e.g., both the PLR and psychosensory–dilation effects), this becomes less viable (Blini et al., 2024a, b). Regular PCA is a purely descriptive method: the eigenvector of PC1 in Fig. 1B has the distinctive PLR shape because the data in Fig. 1A show the distinctive PLR shape. PCA attempts to account for the largest share of the variability in the data, but it is absolutely agnostic as to whether the data themselves contain potentially distinct generators. For example, when, in our recent study (Blini et al., 2024b), we introduced a manipulation of working memory load (WML) on top of changes in luminance of the numbers, the eigenvector of the first component fell precisely midway between those obtained when evaluating the two processes separately. In other words, temporal PCA could not discriminate well between these two processes with known, very different neural generators, but only found a convenient, parsimonious solution to summarize the data, which happened to collapse both dimensions.

To conclude, PCA can represent an appealing inferential approach, capable of effectively summarizing the data and simplifying the analyses. However, interpretation of PCA in functional terms should be limited to instances that we are confident only include one major process (e.g., either the PLR or WML, but not both), and we are interested in gauging their strength. Otherwise, when multiple processes are suspected, one can consider data reduction approaches closer to factor analysis (Cattell, 2012).

### A factor-analytic approach for pupillometry

We start by stating that factor analysis and (rotated) PCA are different methods, though very often confused (Jolliffe & Cadima, 2016) and often converging with enough data points (Revelle, 2024). From factor analysis, some approaches to PCA have borrowed two procedures: the scaling of eigenvectors and rotations. When eigenvectors are scaled (i.e., by the square root of the eigenvalues), they more intuitively reflect the correlations of the original variables (the time points) with the components (i.e., their “loadings”). This, together with rotations, is often preferable for interpretability. Rotations further transform the loadings according to clear predefined objectives and criteria. For example, rotating matrices can be determined so that, e.g., loadings present maximal correlation with one component but minimal with another. Rotations can also loosen some constraints of PCA, such as that components must be orthogonal—the new ones are allowed, in the so-called oblique solutions, to be correlated. All these measures have the advantage of more neatly separating potentially distinct constructs in the low-dimensional, latent space. The components that can be identified in this way very often resemble known theoretical constructs, and the fact that they can potentially correlate is often regarded as more sound for most use cases. For instance, neuropsychological deficits, as seen for example following stroke, tend to manifest in correlated, though independent, clusters: in this case, rotations are usually more effective in identifying these clusters and theoretically separable domains, whereas regular PCA would return a more general “deficit severity” score (e.g., Bisogno et al., 2021; Blini et al., 2025). The only drawback is that, while the overall amount of explained variance is preserved, the new components present a more evenly distributed explanatory power. In other words, rotations can be applied when efficiency in reducing the data is secondary to its functional interpretation in terms of latent constructs.

Rotated PCA is implemented in the package as reduce_rPCA(), a wrapper around psych::principal() (Revelle, 2024). Please note that this function, even in the absence of rotations, produces slightly different results from stats::prcomp() in light of several differences in parametrization and analytical approach (Revelle, 2024). The command is otherwise very similar to that presented above.
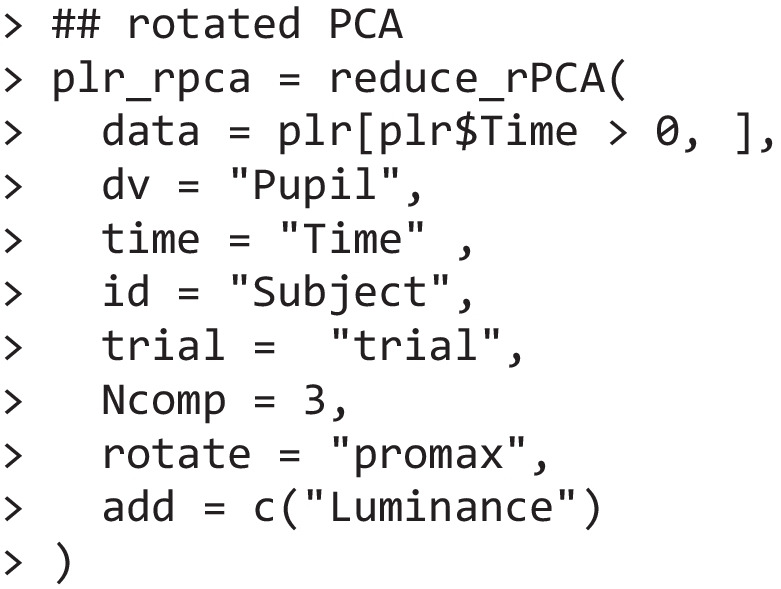


By default, however, reduce_rPCA() applies promax rotation (Hendrickson & White, 1964). The same utility functions seen above can be leveraged as well. For example, the function below depicts the loadings of the three components and the share of variance that they account for, similarly to Fig. 1B.
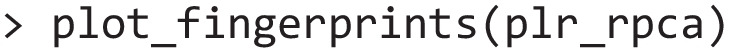


When assessing the scores of these components, all three code for a low-dimensional space in which different luminance levels are well separated. This can be done with the same commands used for temporal PCA. For example, we report below the results for the third rotated component.
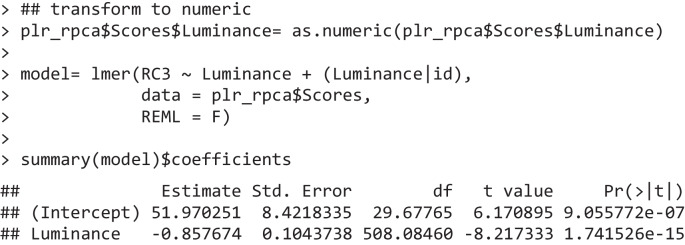


**Fig. 2:**
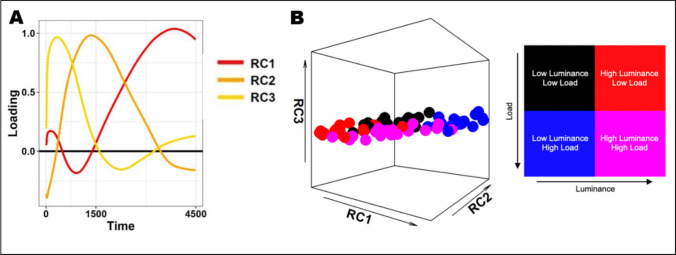
Panel **A** depicts the loadings of the three rotated principal components that we suggest constitute distinct aspects of a pupillary manifold (Blini et al., 2024b). The curves are the average of the loadings obtained (consistently) across three different tasks: one assessing the pupil response to light, one assessing the pupil psychosensory response to working memory load, and one assessing both simultaneously. The loadings were extremely similar, suggesting that, rather than the function measured by the task, they originate from common, “harder” constraints. The suggestion is that these constraints are rooted in physiologically relevant processes (e.g., Steinhauer & Hakerem, 1992; Wetzel et al., 2016; Widmann et al., 2018): sympathetic activation (RC1), parasympathetic inhibition (RC2), and parasympathetic activation (RC3). Scores can be obtained from these loadings to gauge the corresponding process. The scores then define the coordinates on the low-dimensional, latent space that governs phasic changes in pupil size: a pupillary manifold (panel **B**). In panel **B**, each point depicts the average scores of each individual participant, and the color code represents the interaction between luminance and cognitive load as detailed in depth in Blini et al. (2024b)

Thus, assessing all three component scores allows us to conveniently draw inferences about qualitative/quantitative differences in the pupil traces under different light levels. In other words, rotations do not detract from this approach as an *inferential* method. On the other hand, however, one may wonder whether any of these components can be considered as a construct tied *specifically* to the pupillary light reflex. In fact, and even more compelling, these components were identical when promax-rotated PCA was applied to a task assessing working memory load (without changes in luminance) or to a task which varied for both dimensions (Blini et al., 2024b): the same constructs underlay the data in these three very different tasks and their requirements. These loadings, remarkably similar across tasks, are depicted in Fig. 2A**—**we averaged the loadings in the three conditions precisely because they were extremely similar. This finding actually fits very well with previous attempts to apply this procedure to pupil traces obtained from radically different tasks (Bonmassar et al., 2020; Jainta & Baccino, 2010; Wetzel et al., 2016; Widmann et al., 2018; Wong et al., 2020). For example, Wetzel et al. (2016) used promax-rotated PCA to dissect and analyze the pupil dilation response to deviant sounds in an auditory oddball paradigm, in both children and adults. The authors particularly focused on two late components, very similar to RC1 and RC2 depicted in Fig. 2A, even though rotated PCA was intended to retrieve *k* = 3 components. Note that, in contrast to temporal PCA, this parameter may change the outcome of analyses when rotations are involved, because components are not merely defined by explained variance but also by the requirement to maximize interpretability across components.

Altogether, these findings point toward the existence of very general—that is, task-independent—mechanisms and constraints at the roots of these identical components. At a glance, however, we are facing a situation similar to that encountered with temporal PCA, in which the scores were very handy to analyze, but then did not discriminate between different constructs (e.g., PLR, WML). Is there an advantage in using a rotation?

One advantage is precisely the apparent stability of this solution. Across different research groups and tasks of different natures, the shape of the loadings is not idiosyncratic to the specific dataset: rather, results can be reported down to the same, common low-dimensional space. This enables, potentially, an easier comparison of the results but, more importantly, the possibility to refer to the same physiological constructs. The second advantage is that, while the rotated components do not seem to separate different functions—such as the pupil response to light or the psychosensory response to cognitive load—they do bear a striking resemblance to the distinct waves identified by several authors as the signature of key physiological processes (Steinhauer et al., 2004, 2022; Steinhauer & Hakerem, 1992). Indeed, as outlined above, several distinct waves can be identified in the pupillary recordings by selective blockade of pupillary sphincter or dilator muscles as well as manipulation of the environmental light level, and then ascribed to specific physiological processes. Thus, in line with several authors (e.g., Steinhauer & Hakerem, 1992; Wetzel et al., 2016; Widmann et al., 2018), the suggestion is made that the rotated components in Fig. 2A depict and quantify the strength of, in ascending order of peak latency: parasympathetic activation (RC3), parasympathetic inhibition (RC2), and sympathetic activation (RC1). These factors represent the physiologically relevant signals that, altogether, constitute phasic changes in pupil size. It is therefore reassuring that the structures can be identified consistently (1) regardless of the task at hand and (2) regardless of whether they discriminate between different experimental conditions or not. This would be entirely expected if they were based on hard, biological dynamics and constraints rather than a specific function, particularly important for the context. Indeed, in Blini et al. (2024b), the first two components (depicted in red and orange in Fig. 2A) could also discriminate very well different conditions of cognitive load, in addition to the luminance of the stimuli. However, the third component—RC3, i.e., parasympathetic activation—defined a latent space along which only different luminance levels were well discriminated, but not different levels of cognitive load. In other words, RC3 appeared specifically sensitive to changes in luminance, but not mental effort. And yet RC3 was recovered regardless of its role in the task at hand (e.g., also when probing only cognitive load). In other words, the corresponding physiological process, while silent, still unfolds to a significant extent and concurs in explaining the observed changes in pupil size. Assessing the low-dimensional space of changes in pupil size also allows one to account for this “nuisance” aspect.

## Pupillary manifold(s) in perspective

We suggest that the low-dimensional space of phasic changes in pupil size can efficiently represent most physiological variations of interest for cognitive neurosciences. In keeping with contemporary neurosciences (Gallego et al., 2017; Langdon et al., 2023), we have therefore introduced the notion of pupillary manifold (Fig. 2B). A manifold is defined as any space that is locally similar to a Euclidean space (i.e., simple enough) while potentially presenting more complex shapes, properties, and parameters globally. For example, in spite of the overwhelming richness of information provided by neurons within a given population, dimensionality reduction techniques can identify latent spaces in which the same information is represented much more efficiently (e.g., at the population level, rather than at the single-unit level) and at the same time in a much more human-accessible way: a neural manifold. One instance is the coding of head direction (Chaudhuri et al., 2019): the direction of the head can be coded very smoothly on a one-dimensional, ring-shaped manifold, and through one single parameter (i.e., the angle). Likewise, the pupillary manifold would represent all the observed phasic changes in pupil size very efficiently in terms of the strength of the relative autonomic processes, that is, sympathetic and parasympathetic activity. Our suggestion is therefore to assess this manifold in order to retrieve the corresponding coordinates (the scores), with the ultimate goal of obtaining few and physiologically meaningful dependent variables for statistical inference.

The shift toward the analysis of population-level encoding has been a major paradigm change for neurophysiology (Ebitz & Hayden, 2021). The dimensionality of the problem tackled with neuronal recordings is, of course, very vast when compared to the more modest scale of pupillometry data. For this reason, we do not anticipate major breakthroughs with respect to established analytical approaches. In other words, we expect all these approaches to converge to very similar conclusions. On the other hand, however, we find it worthwhile to stress and summarize the potential advantages of assessing the pupillary manifold before outlining future perspectives.

- (1) The first advantage is that the analytical approach would be mindful of the underlying physiology. In other words, the variables used for statistical inference would have a direct counterpart in our principled knowledge of the physiological processes that underlie phasic changes in pupil size. This is not only often more theoretically sound (in the terms outlined in the introduction), but can facilitate the cross-talk between different disciplines (i.e., psychology and neurophysiology) by favoring the use of the same language and the reference to the same constructs. For the purpose of computational modelling, finally, these scores may represent more meaningful, other than efficient, proxy values to be parametrized as latent processes.
- (2) The second advantage is that the approach returns one or very few (e.g., three) values, which makes statistical modelling extremely manageable. Likewise, the corrections for multiple tests are also easier and less strict, because computed on fewer values. These advantages extend to the design of a study, and particularly preregistration and power analysis. It is arguably easier to predict in advance that different conditions will differ in the strength of their “sympathetic activation” rather than predict the specific time window in which differences will be observed, as this may be context-dependent. Likewise, it is also arguably easier to perform a statistical power analysis on these single values, rather than on raw pupillary traces. Importantly, the scores do not lose their “temporal” aspect: they are the product of the temporal information conveyed by the eigenvectors, and reflect the overall alignment of the trial with this structure.
- (3) Third, the approach has the potential to shield us from some arbitrary choices. The approach is fully data-driven and, as such, does help in reducing researchers’ degrees of freedom (Calignano et al., 2024). Of course, while some degrees of freedom are eliminated entirely, others are introduced: How many components should we retain for analyses? Do we consider all loadings, including those close to 0, to compute the scores, or rather introduce a (more or less arbitrary) threshold? What is the best normalization procedure (or lack thereof) prior to PCA? For all these parameters and analytical choices, a consensus will be needed across groups.

The consensus across groups is really warranted, not only for the technical minutiae related to implementing one technique, but also because more fundamental questions remain open and are well worth an answer. The most compelling ones, which we suggest should be the focus of future research, are presented in Box 1.

## Box 1 Outstanding questions

**Table:** 

Methodological questions	• Is there a better dimensionality reduction technique or computational model to describe the pupillary manifold? • What is the most appropriate preprocessing pipeline in terms of, e.g., data normalization? Can a consensus be put forward for the hyperparameters? • Do the physiological insights gathered extend to more complex designs, e.g., dynamic, naturalistic settings?
Theoretical questions	• Can we consistently describe a single pupillary manifold, or rather, do we have evidence of distinct ones? • What is the relationship between the coordinates of the pupillary manifold and the (three) brain networks previously identified through cross-correlations with pupil size (Radetz & Siegel, 2022)? Can we take each score as a proxy for the relative strength of the activation in that network?

### Conclusions

The established (temporal) approaches to deal with pupillometric recordings (e.g., Hershman et al., 2019; Mathôt & Vilotijević, 2023; van Rij et al., 2019) are likely to provide powerful, interpretable results. We refer the readers to these papers for excellent overviews of these methods, which we are confident will serve their purposes well.

What we have described in this tutorial paper is an alternative approach that makes theory-informed use of dimensionality reduction techniques. Temporal PCA can be used to summarize, over the course of a trial, the strength and shape of the dominant signal, so that different conditions can be compared at this level. This represents a powerful inferential approach, though it may not be suited to identifying separable sources within the original signal. Rotated PCA, on the other hand, in addition to offering a powerful inferential approach (to assess differences between conditions), may provide deeper mechanistic (physiological) insights. Both approaches are best suited for strictly controlled (laboratory) settings. Here, it is very common to have designs that can be clearly segmented (“locked,” in electroencephalogram [EEG] terminology) and baseline-corrected into meaningful epochs. More complex designs, and designs that lead to pupil traces of uneven length (e.g., when a response time is involved), may be less suitable for this approach, which may only hold for quick and systematic phasic bursts. However, more studies are needed to pinpoint precisely how dimensionality reduction can be fully exploited. In this tutorial, we have attempted to provide a balanced overview of the rationale and possible advantages that these approaches may bear. We therefore encourage researchers using pupillometry to explore whether dimensionality reduction represents a convenient or otherwise suitable approach for their own research.

To facilitate this aim, we provide a set of open source wrapper functions for dimensionality reduction (e.g., PCA, rotated PCA) implemented in R (R Core Team, 2023):

https://github.com/EBlini/Pupilla

The documentation for the main functions as well as vignettes is available here:

https://eblini.github.io/Pupilla/

## Data Availability

This paper does not include primary data. The original dataset used here as an example is available at the following link: https://osf.io/dkpcs/

## Code availability

The functions described here are available through GitHub:

https://github.com/EBlini/Pupilla

For the documentation, see https://eblini.github.io/Pupilla/
